# CBS: an open platform that integrates predictive methods and epigenetics information to characterize conserved regulatory features in multiple *Drosophila* genomes

**DOI:** 10.1186/1471-2164-13-688

**Published:** 2012-12-10

**Authors:** Enrique Blanco, Montserrat Corominas

**Affiliations:** 1 Departament de Genètica and Institut de Biomedicina (IBUB), Universitat de Barcelona, Av. Diagonal 643, 08028, Barcelona, Spain

**Keywords:** Gene regulation, Genomics, Epigenomics, Comparative genomics, ChIP-seq

## Abstract

**Background:**

Information about the composition of regulatory regions is of great value for designing experiments to functionally characterize gene expression. The multiplicity of available applications to predict transcription factor binding sites in a particular locus contrasts with the substantial computational expertise that is demanded to manipulate them, which may constitute a potential barrier for the experimental community.

**Results:**

CBS (Conserved regulatory Binding Sites, http://compfly.bio.ub.es/CBS) is a public platform of evolutionarily conserved binding sites and enhancers predicted in multiple *Drosophila* genomes that is furnished with published chromatin signatures associated to transcriptionally active regions and other experimental sources of information. The rapid access to this novel body of knowledge through a user-friendly web interface enables non-expert users to identify the binding sequences available for any particular gene, transcription factor, or genome region.

**Conclusions:**

The CBS platform is a powerful resource that provides tools for data mining individual sequences and groups of co-expressed genes with epigenomics information to conduct regulatory screenings in *Drosophila*.

## Background

Massive genome-wide characterization projects have dramatically transformed our current view of genes and other elements of the genome [1]. The picture emerging is of a complex regulatory landscape in which multiple actors coincide to perform distinct roles that are fundamental for the appropriate deployment of cellular gene expression programs [2]. Transcription factors (TFs) are protein adaptors that recognize particular regulatory sequences (TF binding sites, TFBSs) in the genome to target the assembly of other protein complexes that ultimately govern gene expression [3]. In fact, precise information about when and where a gene is transcribed is encoded on the sequence and the structure of the genome.

At the sequence level, promoters are regulatory regions located immediately upstream of the gene, which anchor the RNA polymerase transcriptional machinery to the transcription start site (TSS), while enhancers conduct more precise tissue-specific gene expression and can be physically displaced up to hundreds of kilobases from their target. Both promoters and enhancers are non-coding sequences in which multiple TFBSs of about 5 to 15 bp are distributed following a modular organization. Such cis-regulatory modules (CRMs) act as genetic switches and are bound by specific TFs to drive distinct patterns of expression. Comparison of binding landscapes across multiple species have revealed that these functional regulatory regions tend to be highly conserved throughout evolution in many cases [4-6]. The predominant model establishes that direct contact between both enhancers, promoters and TFs, through DNA looping orchestrates the RNA polymerase recruitment to initiate the transcription of the neighbouring gene [7].

At the structural level, chromatin packaging into nucleosomes dynamically shapes the genome, producing a landscape of open and closed regions that can eventually show or mask different pieces of information encoded within the sequence [8]. By interpreting a collection of post-translational modifications of the histone tails at the surface of nucleosomes, chromatin remodeling complexes can force a repositioning of such structural units, resulting in a change in the local conformation of a particular area [9]. Consequentially, modifications in the chromatin structure may confine access of TFs to a subset of regulatory sequences along the genome [10]. Recent studies on epigenomics have unveiled the existence of chromatin signatures that are helpful to distinguish promoters and enhancers from other genome elements [11-13]. Thus, while active gene promoters are in general marked by trimethylation of lys4 of histone H3 (H3K4Me3), distal enhancers are associated with monomethylation (H3K4Me1). However, functional enhancers for active genes exhibit additional acetylation of lys27 of histone H3 (H3K27Ac), while trimethylation of lys27 (H3K27Me3) denotes poised enhancers that are linked to inactive genes [11,13].

Deciphering the map of regulatory sites and regions that shape the genome is therefore a formidable challenge of major interest, for which computational methods that identify such features can be extremely helpful. Most bioinformatics protocols for regulatory prediction consist in the application of two steps (reviewed in [14-16]): (i) sequence analysis in search of consensus sites derived from catalogs of predictive models or motif discovery approaches; and (ii) evaluation of such predictions, taking into account evolutionary conservation across other species. Recently, additional epigenomic information about histone modification maps has been integrated into other approaches, and this significantly outperforms previous strategies [17-19]. In the last decade, a myriad of bioinformatics solutions have been published that deal with the problem of mapping putative TF sites and predicting regulatory regions (see [20] for a comprehensive listing). As a consequence, scientists must face a plethora of heterogeneous tools in order to characterize a regulatory region, including, among others, genome browsers [21,22], multiple genome alignments [23,24], catalogs of functional sites [25-27], software suites of prediction [28,29], and genome-wide epigenetics profiles [30,31]. Even though unquestionable progress is observed in this issue, through integrative initiatives such as Galaxy [32], this complex mixture of applications and databases often constitutes an obstacle for basic researchers, who are actually the potential target audience demanding this information. The minimal computational expertise that is required may be prohibitive for many experimentalists, denying them access to this knowledge that could expedite their investigations at the lab bench [33].

Research on transcriptional regulation in *Drosophila melanogaster*, one of the most intensively studied organisms in biology, is a case in point. In fact, the sequencing of other flies [24] offers a formidable opportunity to decipher the common regulatory circuitry of these species. This information is fundamental when conducting experimental research to elucidate potential relationships between regulators and their targets [34]. More recently, the modENCODE project has released more than 700 genome-wide datasets for dozens of TFs, histone marks, and other regulatory features that promise to drastically push the field of characterization of *Drosophila* gene regulatory regions forward in the next decade [35]. By and large, fly researchers can work with many resources that provide inestimable access to such information: FlyBase is the major repository of genetic and genomic information on the fruit fly [36], FlyMine is a web platform that integrates external genomics and proteomics resources under the same query interface [37], and modMine provides access to modENCODE data [31]. Specifically for the characterization of TF binding sites, the information is distributed into different resources: FlyTF is a comprehensive catalog of TFs with DNA-binding properties [38], while REDfly, FlyFactorSurvey, and the DNase footprint database are compilations of TFBSs experimentally validated in *Drosophila*[39-41]. Moreover, Jaspar and Transfac repositories include about 100 predictive models derived from the literature for *Drosophila*[25,26].

However, although important efforts are being done to standardize the construction of large-scale collections of regulatory sites [6,42], it is not trivial for a bench biologist to understand how to deal with this massive volume of information (for examples, see [43,44]). As a result, there is a need for easy-to-use web integrative resources that perform comparative regulatory analyses on emerging next-generation sequencing data. We present here CBS (Conserved regulatory Binding Sites), an open regulatory platform that offers, under an intuitive graphical interface, a comprehensive map of evolutionarily conserved binding sites and enhancers identified in *Drosophila*, using a combination of predictive and alignment methods with epigenomic information. Through the introduction of user custom tracks for most popular genome browsers, CBS makes visualization of these regulatory features extremely simple for inexpert users. We demonstrate how CBS can be particularly useful for characterizing functional sequences and conducting *in silico* regulatory screenings of target genes reported in high-throughput expression experiments.

## Implementation

### Prediction of conserved TFBSs

CBS integrates regulatory information for 21,984 RefSeq transcripts [45] of 13,678 genes from *D. melanogaster* (BDGP Release 5/dm3 April 2006). Prior to computational prediction of TFBSs and enhancers, we masked the sequence of all known exons using current gene annotations in RefSeq [46]. We constructed a catalog of 850 predictive models of TFs that integrates 255 weight matrices from Jaspar CORE [25] and 595 weight matrices from Transfac 8.4 [26]. For simplicity, users see the full catalog of matrices organized into 346 distinct TFs, whereby each one may represent occurrences for several matrices that predict the same class of TF. To produce the initial set of predictions, we searched for best occurrences of each predictive matrix on the fruit fly genome with MatScan [47], discarding those hits with a similarity below 85% of each weight matrix. The phastCons program computes conservation scores based on a phylo-HMM with two states (conserved and non-conserved regions) that correspond to the posterior probability that a given alignment column is generated by the conserved state of the phylo-HMM [48]. From the initial pool of predictions, we removed those binding sites within genome regions in the UCSC genome browser [21] that presented on average a probability lower than 0.95 to be conserved across the 12 flies multiz15way alignments [49]. This treatment resulted in a five-fold reduction of binding sites. Users can instruct CBS to include predictions that exhibit a probability higher than 0.95 to be in conserved regions (moderate), or force hits that present a probability of 1 (maximum) to be displayed. All predictions, irrespectively of the conservation score, can be downloaded from CBS website as flat files.

### Prediction of putative regulatory enhancers

We gathered ChIP-seq enriched regions for H3K4Me1, H3K4Me3, H3K27Ac, and H3K27Me3, as reported by the modENCODE consortium [35,50], in the following developmental stages: embryos (0–4 h, 4–8 h, 8–12 h, 12–16 h, 16–20 h, and 20–24 h), larvae (L1, L2, and L3), pupae, and adult males and females (see Additional file 1 for further details). We considered each non-coding genome region that presented a significant H3K4Me1 signal to be a putative enhancer. To separate enhancers from gene promoters, we generated an alternative list of regions exhibiting enrichment in H3K4Me1 that lacked the H3K4Me3 signal. To distinguish active enhancers from poised enhancers, we searched for those genome regions that showed enrichment either in H3K27Ac or H3K27Me3 in the same developmental stage as well (Additional file 2). To focus our search on non-coding sequences (intergenic and intronic regions), we masked previous predictions when overlapping RefSeq exons (Additional file 3). We provide access for graphical display in the CBS website to a subset of these predictions: we discarded those putative enhancers that showed a probability lower than 0.5 to be conserved in 12 flies multiz15way alignments produced by the UCSC Genome Browser [23,24], removing shorter regions (less than 200 bp) that might produce artifacts from this procedure. Following this protocol over all stages, we ended up with 15,454 putative enhancers (regions with at least H3K4Me1, average size 985.1 bp), 13,326 putative enhancers excluding gene promoters (regions with H3K4Me1 that lack of H3K4Me3, average size 985.3 bp), 6 269 active enhancers (regions with H3K4Me1 and H3K27Ac, average size 936.8 bp) and 4 847 poised enhancers (regions with H3K4Me1 and H3K27Me3, average size 1215.7 bp). In summary, our set of predicted enhancers roughly involves 8% of the fruit fly genome in terms of total coverage.

### Data sets from other *Drosophila* genomes

From available pairwise BLAT alignments between *D. melanogaster* RefSeq genes and the rest of *Drosophilids* available in the UCSC genome browser (xenoRefGene tracks), we constructed a putative gene annotation for the following assemblies: *D. ananassae* (droAna1), *D. erecta* (droEre1), *D. grimshawi* (droGri1), *D. mojavensis* (droMoj2), *D. persimilis* (droPer1), *D. pseudoobscura* (dp3), *D. sechellia* (droSec1), *D. simulans* (droSim1), *D. virilis* (droVir2), and *D. yakuba* (droYak2)*.* In previous works [51,52], we extracted 1 000 nucleotides upstream of the TSS to define gene promoters. Here we took a more conservative approach and considered the region within 2 000 nucleotides upstream of the corresponding TSS when analyzing multiple promoters for 11 *Drosophila* genomes. MatScan [47] is used to search for best occurrences of a particular TF on each set of orthologs. The GFF2PS program [53] produces the graphical map of final predictions.

### Supporting information

From REDfly v3.2 [39], we gathered 1 830 experimentally validated CRMs, and 1 825 TFBSs reported in the literature. Moreover, we imported the binding instances for 56 TF motifs with conservation confidence values of 60% or higher as identified by Kheradpour *et al.*[6].

### Implementation details

CBS web interface is implemented via a set of PHP scripts to provide access for different MySQL tables that store gene annotations, collections of predictive models, predicted TFBSs/enhancers, and orthologous promoters in multiple species. All these catalogs are publicly distributed as stand-alone flat files at the CBS website. Configurable balloon tooltips introduced in CBS help menus were designed by Sheldon McKay in the Cold Spring Harbor Laboratory for the Generic genome browser [54].

## Results and discussion

### Database features

#### Content

**Figure 1:**
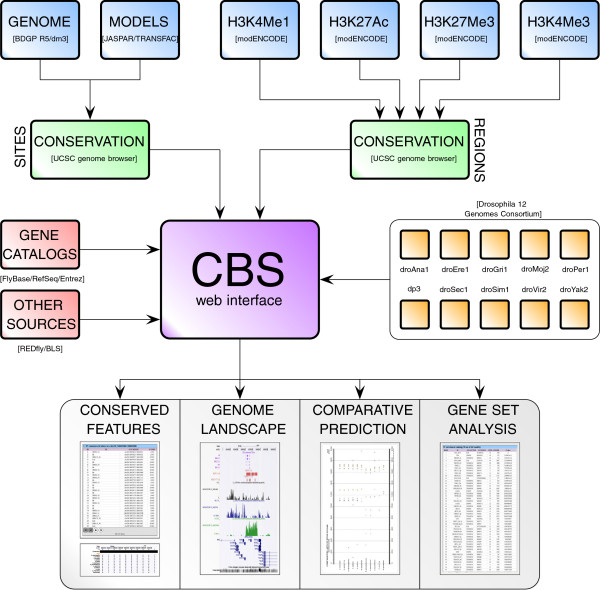
**Workflow of the CBS analysis platform.** CBS functions can be divided into two categories of evolutionarily conserved information: prediction of regulatory sites, and identification of putative enhancers. The CBS website interface integrates both classes of predictions with other regulatory resources, such as experimentally validated sites and genome-wide profiles of several histone post-transcriptional modifications, in order to characterize *in silico* a region of the *Drosophila* genome.

CBS reports a body of predictions and experimental evidence from several sources of information that are combined using a bioinformatics protocol aimed at substantially masking the complexity of such tasks (see Figure 1 and Table 1). For virtually each non-coding region in the fruit fly genome, users can call up the following information (see Implementation): (a) computationally predicted 27,868,614 sites for 346 TFs that are phylogenetically conserved along multiple *Drosophilids*; (b) 15,454 putative enhancers (6 269 active and 4 847 poised enhancers), computationally inferred from modENCODE ChIP-Seq data [35] throughout all developmental stages; (c) 1 830 CRMs and 1 825 TFBSs gathered from experimental literature in the REDfly database v3.2 [39]; (d) 52,724 computational binding sites predicted by Kheradpour and colleagues [6]; (e) genome-wide ChIP-seq profiles for H3K4Me1, H3K4Me3, H3K27Ac, and H3K27Me3 from modENCODE [35] for all developmental stages; and (f) computational predictions for 346 TFs on 22,763 orthologous promoters of RefSeq genes from 10 *Drosophila* genomes [21]. CBS functions can be interrogated using multiple equivalent gene name nomenclatures from distinct sources, such as FlyBase, RefSeq, NCBI Entrez, Gene symbol names, and CG codes.

**Table 1:** Summary of data sources integrated into CBS

**Information**	**Source**	**Reference**
*D. melanogaster* genome (BDGP R5/dm3)	UCSC Genome browser	[21]
*D. melanogaster* gene catalog	FlyBase	[36]
*D. melanogaster* gene catalog	RefSeq	[45]
*D. melanogaster* gene catalog	NCBI Entrez	[55]
Predictive models	JASPAR	[25]
Predictive models	TRANSFAC	[26]
H3K4Me1 ChIP-Seq profile	modENCODE	[35,50]
H3K4Me3 ChIP-Seq profile	modENCODE	[35,50]
H3K27Ac ChIP-Seq profile	modENCODE	[35,50]
H3K27Me3 ChIP-Seq profile	modENCODE	[35,50]
Conservation scores	UCSC Genome browser	[21]
Experimentally validated CRMs	REDfly	[39]
BLS predictions	BLS	[6]
*D. ananassae* genome	UCSC Genome browser	[21]
*D. erecta* genome	UCSC Genome browser	[21]
*D. grimshawi* genome	UCSC Genome browser	[21]
*D. mojavensis* genome	UCSC Genome browser	[21]
*D. persimilis* genome	UCSC Genome browser	[21]
*D. pseudoobscura* genome	UCSC Genome browser	[21]
*D. sechelia* genome	UCSC Genome browser	[21]
*D. simulans* genome	UCSC Genome browser	[21]
*D. virilis* genome	UCSC Genome browser	[21]
*D. yakuba* genome	UCSC Genome browser	[21]
Pairwise comparisons	UCSC / RefSeq	[21,45]

#### User interface

**Figure 2:**
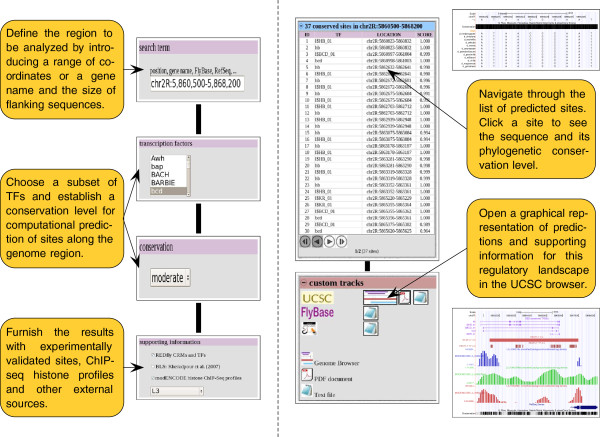
**Basic usage of the CBS interface.** Input information that is necessary to interrogate the system in search of predictions and annotations on a particular locus (left). Output provided for the characterization of a genome region in terms of TF binding sites of a certain family (right). Such data is divided into a list of individual sites, and a global representation of these predictions is visualized in a genome browser.

The CBS website is designed to minimize the number of interactive steps that users should follow to end up with the display of the resulting information, offering a common interface to perform each analysis protocol on a particular input set. In consequence, the basic usage of this tool generally requires four fundamental elements (see Figure 2, left): (i) information regarding the locus to be analyzed (gene names or genome coordinates); (ii) class of TFs in which the user is interested; (iii) conservation level between species that is expected for final predictions; and (iv) supporting information from external sources (REDfly [39], BLS [6] and modENCODE histone ChIP-seq profiles [35]) that must be integrated into the final output. Users will find abundant help in the website on the particular options of each CBS function, together with hands-on tutorials that include suggestions about the interpretation of the results.

**Figure 3:**
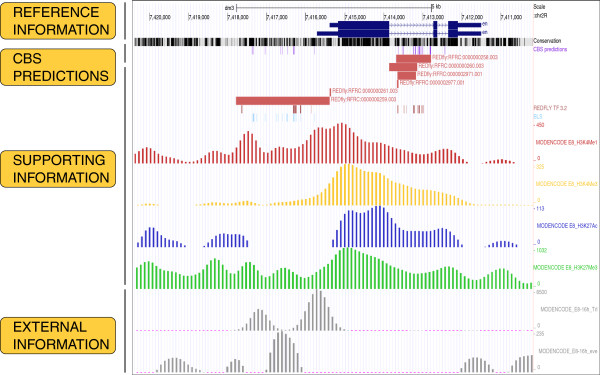
**Visualization of CBS information on UCSC genome browser.** This example illustrates the graphical display of the global output representation. From top to bottom, the following tracks are shown: RefSeq *en* gene, UCSC conservation between *Drosophila* genomes [21], CBS predictions, REDfly experimental CRMs [39], BLS predictions [6], and modENCODE ChIP-seq profiles for chromatin marks associated to enhancer sequences [50]. As external information, we manually incorporated in this case two custom tracks in grey color for Trl and Eve binding profiles as published by modENCODE [50] .

CBS outputs are presented following a similar layout. Thus, results for a query are divided into three main blocks (see Figure 2, right): information about the options selected by the user in previous screens and genes involved in current analysis, the list of individual TFBSs that are predicted in regions of the fruit fly genome that present a significant conservation level among *Drosophilids*, and the files of custom tracks to produce global representations of the results in several genome browsers as UCSC [21], FlyBase [36], and modENCODE [31]. Knowledge about such resources is graphically presented as independent tracks of annotations that users can easily show or hide, to facilitate the visibility of the final picture [56]. CBS exports the information solicited by the user to different genome browsers, taking advantage of their standard interface. For example, by clicking on the location of a particular site in the list of individual predictions, its sequence can be accessed in the genome using the UCSC genome browser. On the other hand, when it is necessary to visualize the global regulatory landscape of the region that is currently analyzed, users can integrate both predictions and supporting information into a single file following conventional genome browsing standards, by clicking on the graphics icon of this section (see Figure 2, right). As a result, it is feasible to represent TFBSs and enhancers predicted by CBS, together with experimental data and phylogenetic conservation status on the UCSC genome browser framework (Figure 3). A similar graphical representation can be obtained for Gbrowse servers such as FlyBase and modENCODE (see Additional file 4). All these tools permit the incorporation of results from other high-throughput experiments performed by the user, or from external data from the literature, into the final picture for posterior integrative analysis [56].

### Applications

#### Characterizing genome regions and gene locus

Frequently, wet-lab biologists suspect that a particular TF might participate in the transcriptional regulation of a gene of interest. To address this question, CBS automatically produces the map of predicted TFBSs, demanding a certain conservation level in multiple *Drosophilids*, and includes epigenetic information into the results to favor the selection of regions that show epigenomics patterns characteristic of enhancers. Therefore, users obtain a list of promising predicted sites at a particular locus together with available experimental information and ChIP-seq profiles for a certain developmental stage. This information can be helpful to discriminate which TFs are feasible as regulators of specific genes, and to find the most solid predictions along the established regulatory region.

As an example, we show the CBS regulatory landscape with predictions for several TFs that are known to be involved in the regulation of the *engrailed* (*en*) gene locus (Figure 3). According to REDfly [39], several CRMs have been experimentally reported in the gene promoter and its first intron. We additionally observed enrichment from ChIP-seq results for H3K4Me1, H3K27Ac, and H3K27Me3 from modENCODE [50] in embryos (between 8 h and 12 h). This information can be extremely useful in many occasions to decide which predictions are more reliable. To further highlight the potential of this approach, we manually imported ChIP-seq binding profiles of Trl (GAF) and Eve from embryos (E8—16 h; modENCODE ID 3397 and 3401, respectively) reported by the modENCODE consortium [50] into the same picture (see Figure 3). With this regulatory map, users can accurately decide which potential sites might be more appropriate for validation. In addition, we offer the option to analyze a group of gene promoters to search for the abundance of a particular class of TFs that might explain similar regulatory patterns. For instance, CBS is able to identify within a group of co-regulated genes reported to be relevant for wing imaginal disc regeneration a significant enrichment on AP1 binding sites (see Table 2), which is a downstream target of the JNK signaling pathway that is precisely activated in wound healing stages [51]. Such results confirm the initial regulatory characterization and incorporate additional predictions that enrich the original description.

**Table 2:** **Classification with CBS of TF binding motifs that are enriched in promoters of 48 class III genes reported to be relevant for wing imaginal disc regeneration**[51]

**Rank**	**Model**	**Source**	**Genes**	**Genome**	***P *Value**
1	**AP1**	BLS	14	838	0
2	TBP	JASPAR	10	573	2.26e-12
3	V$TATA_01	TRANSFAC	10	573	1.55e-11
4	CrebA	BLS	10	1007	1.21e-06
5	V$CDXA_01	TRANSFAC	30	5884	2.97e-06
6	**V$AP1_C**	TRANSFAC	8	746	5.41e-06
7	**V$AP1_Q2**	TRANSFAC	8	704	7.93e-06
8	V$ELF1_Q6	TRANSFAC	5	358	1.46e-05
9	**V$AP1_Q6**	TRANSFAC	8	811	1.93e-05
10	I$GRH_01	TRANSFAC	13	1721	2.06e-05
11	TATA	BLS	5	377	2.28e-05
12	V$USF_Q2	TRANSFAC	5	410	3.70e-05
13	**V$AP1_Q4**	TRANSFAC	6	551	4.96e-05
14	V$CDXA_02	TRANSFAC	32	7214	6.85e-05
15	V$STAT5A_04	TRANSFAC	29	6281	8.48e-05
16	GATA2	JASPAR	33	7698	1.10e-04
17	V$PAX2_02	TRANSFAC	29	6446	1.61e-04
18	V$TBP_Q6	TRANSFAC	24	5032	2.53e-04
19	ct	JASPAR	19	3562	3.92e-04
20	V$GATA1_05	TRANSFAC	7	803	4.51e-04

For each predictive model, the following attributes are shown: rank, name of the model, regulatory catalog, gene promoters in which such a motif is detected, number of gene promoters in the whole genome in which the same motif is identified under the same constraints, and P value. Here we show only the best 20 motifs reported by CBS (see http://compfly.bio.ub.es/CBS/listTF_CBS.php for further details on each predictive model).

#### Exploring putative enhancers

**Figure 4:**
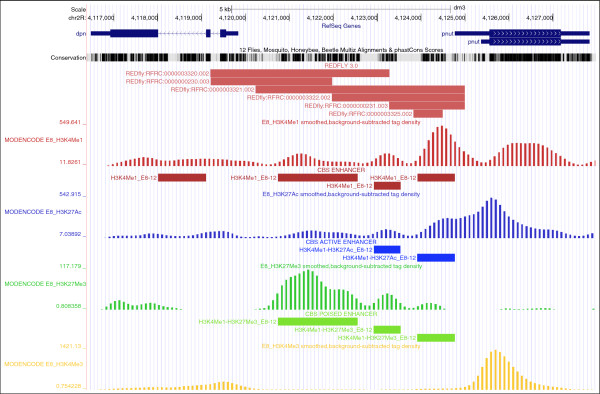
**Prediction of putative enhancers in the fruit fly genome with CBS.** Here we show the *dpn* gene locus in which REDfly annotated six CRMs (shown in red); CBS reported several active and poised enhancers (shown in blue and green, respectively) that overlap with the experimental annotations from 8–12 h–old embryos, according to modENCODE chromatin profiles.

**Figure 5:**
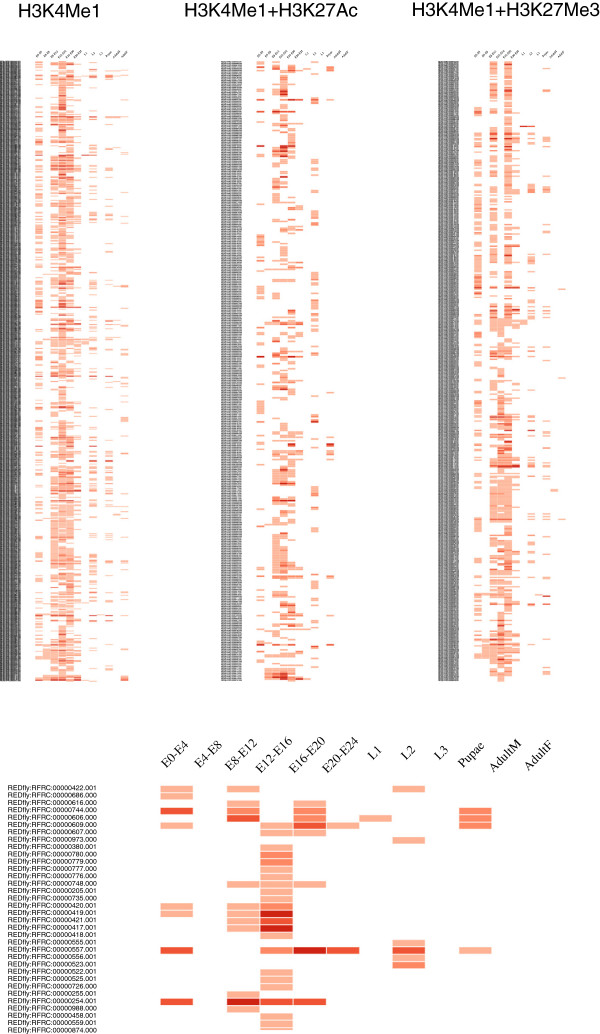
**A heatmap of REDfly CRMs annotated with the catalog of putative enhancers provided in CBS.** At the top are the chromatin signatures of REDfly entries [39] that can be associated with CBS H3K4Me1 enhancers, active enhancers, and poised enhancers, respectively. At the bottom, a magnification of the active enhancer map is shown to illustrate the distinct patterns of developmental stages for a fraction of these CRMs. The darker the intensity of the red color, the higher the number of putative enhancers overlapping a particular CRM. Please note that CBS does not directly produce this type of representation.

**Figure 6:**
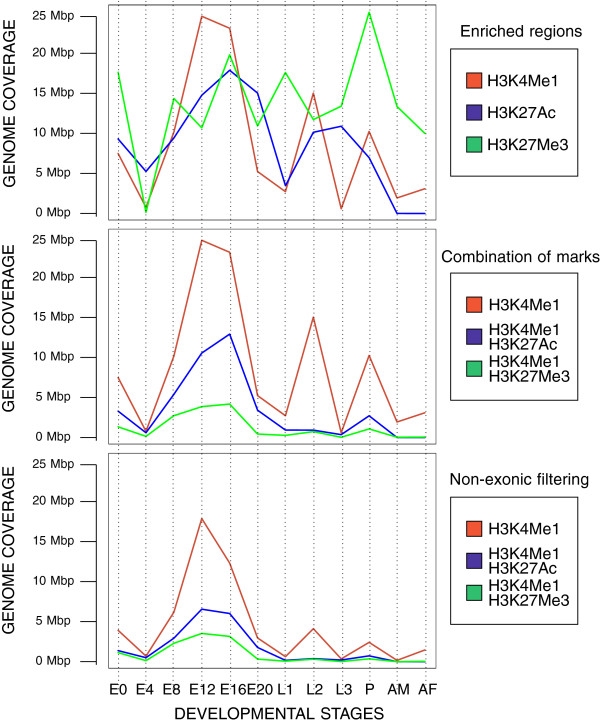
**Genome coverage of each combination of modENCODE histone profiles to identify putative enhancers throughout all developmental stages of the fruit fly.** (First pannel) Distribution of chromatin signatures as provided by modENCODE [50]. (Middle pannel) Distribution of H3K4Me1 and each intersection with a second histone mark. (Last pannel) Distribution of H3K4Me1 and each intersection with a second histone mark when hits overlapping with RefSeq exons were removed.

From modENCODE ChIP-seq profiles for several histone marks studied during *Drosophila* development [50], we built a catalog of potential enhancers —active and poised— that present epigenetics features (see Implementation). For a particular region defined by the user, it is possible to explore the list of CBS predictions of binding sites that are located within potential enhancers in the vicinity of the gene of interest. For example, we show in Figure 4 the upstream promoter region of the *dpn* gene in which CBS reports the existence of evolutionarily conserved active and poised enhancers using modENCODE ChIP-seq data for embryos (8–12 h) that overlap with REDfly experimental annotations. This set of CBS enhancers is freely available from the website and can be used to annotate a particular genome region in order to explore its transcriptional regulation patterns (Additional file 5). To teach readers about the potential of such predictions, we have exemplified them in two scenarios:

(a) Epigenomics characterization of a set of regulatory sequences. CBS putative enhancers can be extremely useful for studying changes in the activation of regulatory sequences throughout development. Thus, users can take advantage of this information to virtually reproduce these patterns on a particular data set. In Figure 5, we propose to explore how the full collection of REDfly CRMs [39] exhibit different epigenomics features along distinct developmental stages. Here, we have graphically represented this information on a heatmap in which the presence/absence of any of the chromatin signatures available in CBS for each individual regulatory region is denoted in red. To study which CRMs present equivalent regulatory patterns, posterior clustering analysis might be introduced on the resulting pictures. We noticed that for these sequences, such epigenomics signals are likewise more abundant in embryonic stages, which fits well with the fact that REDfly is biased towards embryonic enhancers (because most experiments in the literature are focused on that developmental stage). To study in detail this issue, we analyzed H3K4Me1, H3K27Ac, and H3K27Me3 ChIP-seq–enriched regions from modENCODE stratified by developmental state, in which a similar bias is not expected. In all the scenarios we studied (individual marks, combination of marks, and non-exonic filtering of ChIP regions), we consistently found that H3K4Me1 significantly peaked at embryonic stages (Figure 6). This indicates that most regulatory activity takes place at first developmental stages as previously suggested [57].

(b) Identification of putative enhancers in gene-free regions on the fruit fly genome. We consider that our set of predictions can assist in the characterization of novel regulatory regions. Gene-free regions in *Drosophila* genomes are a case in point. We have detected up to 48 regions of at least 50 Kbp in the fruit fly genome that lack RefSeq transcripts. We characterized the chromatin signatures of these in order to explore putative enhancers. In 20 of the 48 regions (42%, P value < 10^-5^), we were able to identify at least one evolutionarily conserved enhancer (see Table 3). In most cases, these regions do not exhibit enrichments of the characteristic H3K4Me3 promoter mark, confirming the absence of active genes within these sequences. In addition, it was possible to report changes in the same predictions between different developmental stages as well (Additional file 6). The annotation of such novel elements might be refined with the mapping of CBS predictions of TFBSs within each one of these regions.

**Table 3:** List of 20 gene-free regions along the genome that contain one or more putative enhancers, as reported by CBS

**ID**	**Coordinates**
1	chr2L:12850000-12900000
2	chr2R:1750000-1800000
3	chr2R:10950000-11000000
4	chr2R:15900000-15950000
5	chr2R:16250000-16300000
6	chr3L:6400000-6450000
7	chr3L:6800000-6850000
8	chr3L:6850000-6900000
9	chr3L:10350000-10400000
10	chr3L:10700000-10750000
11	chr3L:15750000-15800000
12	chr3L:18300000-18350000
13	chr3R:850000-900000
14	chr3R:10750000-10800000
15	chr3R:11400000-11450000
16	chr3R:19250000-19300000
17	chr3R:25150000-25200000
18	chrX:3900000-3950000
19	chrX:7350000-7400000
20	chrX:16050000-16100000

#### Performing comparative genomics

Genome sequencing of multiple *Drosophila* species finished in 2007 provided a huge volume of data that still remains to be explored [24], and important efforts are conducted to improve the annotation of such genomes [31,36]. Comparative analysis using *D. melanogaster*, for which more accurate information is available, can be indeed very effective. A precise sequence comparison method is necessary for the success of phylogenetic footprinting searches in gene regulatory sequences [58]. It is possible, though, that information about the sequence is scarce or even corrupted in certain regions of the genome, which affects the quality of the final alignment. To prevent these problems and provide an alternative comparative method, we have constructed a compilation of orthologous promoters in all species. Therefore, CBS is able to elaborate simultaneously the map of predictions along the promoter of a particular gene in up to 11 *Drosophila* genomes (see Implementation).

**Figure 7:**
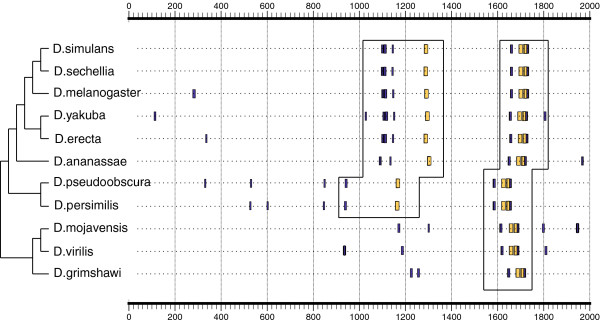
**Comparative map of predictions along the promoter of *E(spl) *in 11 species of *Drosophila.*** TSS is annotated at position 2 000 of each sequence. Predicted sites for Su(H) are depicted in yellow, and predicted sites for E-box motifs are indicated in blue. Two putative regulatory modules containing the Su(H) + E-box composite are denoted with a black square. The phylogenetic tree was manually incorporated into the picture provided by CBS to show the evolutionary distance among species.

To show the utility of this tool, we have focused the analysis on the promoter of the *E(spl)* gene. During *Drosophila* development, the Notch signaling pathway through Su(H) is thought to upregulate the expression of *E(spl)*. When analyzing this sequence in 11 *Drosophila* genomes, CBS uncovered two regulatory modules constituted of Su(H) + E-box sites that are conserved along the gene promoter (see Figure 7). The first module, located around the positions 1 600–1 800, is conserved in all species, in accordance with prior publications [59]. On the other hand, we found a novel second cluster of sites upstream of the first one, which is partially conserved (note that *D. mojavensis*, *D virilis,* and *D. grimshawi* do not show the same arrangement). Although further work is necessary to solidify this prediction, we believe this example illustrates how CBS can be useful in reconstructing the regulatory signature of other genes in these species.

### Quality assessment of predictions

We initiated a procedure of evaluation of the quality of CBS predictions based on the study of how variations in the conservation level of sequences may affect the accuracy of CBS predictions. However, it is important to take into account multiple factors that can influence a potential assessment of such data: (i) experimental evidence may be relevant only for a particular developmental stage or tissue, while most promising computational predictions might actually reconstruct the full map of binding affinities in multiple scenarios; (ii) the quality of the predictive models introduced into this analysis may affect the amount of false positives, which will be different for each TF; (iii) published consensus sequences for TFs are constructed for a few examples, while high-throughput experiments can uncover novel sequences that will not be recognized using current predictive models; and (iv) whether a genome region is enriched in a ChIP-seq signal or not in comparison to a certain control is decided by peak-calling software, which can be configured to be more or less strict, producing variations in the sets of annotated binding regions to be used in the evaluation.

For the aforementioned reasons, we consider that a general assessment of multiple TFs using experimental data is beyond the scope of this work. Thus, we focused our study on particular modENCODE ChIP-seq binding regions for several TFs [50] – Trl (E16–24 h, modENCODE ID 3238), h (E0–8 h, modENCODE ID 2574), ttk (E0–12 h, modENCODE ID 615), sens (E4–8 h, modENCODE ID 2577) – and in modENCODE ChIP-seq hotspots (loci with higher levels of TF binding activity). Sequence conservation along ChIP-seq binding sites of each TF presents heterogeneous patterns, ranging from 0.63 (sens) to 0.34 (ttk) on average (see Additional file 7). In this scenario, we decided to study in detail one of these TFs (GAF/Trl) to learn about conservation and quality of predictions. In this case, when plotting the distribution of Trl true positive real binding sites against the total number of computational predictions, the curve reaches the maximum deviation from the random distribution between 0.40–0.60 (see Additional file 8, top). However, it is interesting to mention that when computing the ratio between the total number of predictions along different conservation levels and the number of successfully identified ChIP-seq binding sites, we observed better predictive values for higher sequence conservation (see Additional file 8, bottom). Nonetheless, taking into account our previous considerations, we would like to emphasize that these results cannot necessarily be extrapolated to other TFs in other developmental stages. Although it is important to capture each class of evidence for each TF and gene regulatory region, we offer only those binding sites that show strong evolutionarily conservation in the CBS website to avoid excessive exposition of information. Those predictions normally fit better with experimentally validated sites (see matches between CBS predictions and published binding sites on *even-skipped* gene stripe 2 enhancer in Additional file 9). For further analysis of the whole body of predictions, we recommend that more expert users download the complete set of predictions for all TFs along the fruit fly genome, which is available as standard GFF files from the CBS website.

To evaluate the accuracy of enhancer predictions, we took into account the collection of 1 830 experimentally validated CRMs published in REDfly v3.2 [39]. To circumvent the overlap between REDfly annotations along the genome, we merged coincident sequences to produce a data set of 726 non-overlapping regulatory regions. When mapping the relationship on the fruit fly genome between merged REDfly CRMs and our set of predictions in any developmental stage, we obtained the following results (*P* value is 0 in all cases): 597 CRMs (82%) present putative enhancer marks (at least H3K4Me1), 357 CRMs (49%) present active enhancer marks (H3K4Me1 and H3K27Ac), and 325 CRMs (45%) present poised enhancer marks (H3K4Me1 and H3K27Me3). In order to establish the importance of sequence conservation, we repeated the assessment using only those predicted enhancers of each class that exhibit higher sequence conservation (UCSC multiz15way alignments with an average score of 0.50 or more), which improved previous results in most cases (*P* value is significant in all cases): 424 of 597 CRMs (71%) confirmed by putative enhancers, 227 of 357 CRMs (64%) confirmed by active enhancers, and 248 of 325 CRMs (76%) confirmed by poised enhancers. In contrast, only 92 CRMs (13%) were associated to putative enhancers when this search was reproduced with predictions that exhibit weak sequence conservation among *Drosophila* genomes.

### Comparison with other tools

A quick exploration of recent literature in search of computational tools to annotate regulatory regions can produce dozens of positive hits. In fact, a deluge of effective bioinformatics approaches have been published that aim to make the computational prediction of TFBSs easier for non-expert users (see for further review [20,60,61]). Although it is beyond the scope of this work to evaluate the performance of each predictor on standard data sets (see [62] and [63] for exhaustive evaluation on motif finding and promoter identification, respectively), we have annotated several features of the most popular tools as compared to our approach. From the different attributes that characterize these applications, we will focus our discussion on the following ones: (i) availability of epigenomics information; (ii) evaluation of predictions with phylogenetic footprinting data; (iii) classes of sequences that can be studied; and (iv) prediction through a web service (see Table 4 for the full list of applications and features).

**Table 4:** List of the most popular computational tools to characterize gene regulatory regions

**Name**	**Species**	**Web Site**	**Genome Regions**	**TFs**	**Comparative Genomics**	**Chromatin Marks**	**Graphical Display**	**Reference**
CBS	12 *Drosophilas*	YES	Full genome	J/T	phastCons	H3K4Me1	UCSC	This work
					BLAT	H3K4Me3	Gbrowse	
						H3K27Ac		
						H3K27Me3		
Chromia	Mouse	YES	Promoters	J/T	NO	H3K4Me1	Own system	[64]
			Enhancers			H3K4Me2		
						H3K4Me3		
						H3K9Me3		
						H3K27Me3		
						H3K20Me3		
						H3K36Me3		
CENTIPEDE	Human	NO	Full genome	J/T	PhyloP	H3K4Me1	UCSC	[18]
						H3K4Me2		
						H3K4Me3		
						H3K9Ac		
						H3K27Ac		
						H3K27Me3		
						H3K20Me1		
BLS	12 *Drosophilas*	NO	Full genome	J/T/ FlyReg	Branch Length Score	NO	NO	[6]
TFMexplorer	Human	YES	Promoters	J/T	NO	NO	Own system	[65]
	Mouse							
	Rat							
	Chicken							
	*Drosophila*							
	*melanogaster*							
DoOPSearch	Multiple	YES	Promoters	-	DoOP	NO	Own system	[66]
	chordates							
	and plants							
COTRASIF	Multiple	YES	Promoters	J/T	Ensembl	NO	Own system	[67]
	vertebrates							
	and plants							
Genome surveyor	*Drosophila*	YES	Full genome		Stubb	NO	Gbrowse	[68]
	*melanogaster*							
MAPPER	Human	YES	Promoters	J/T	NO	NO	UCSC	[69]
	Mouse							
	*Drosophila*							
	*melanogaster*							
Core_TF	Human	YES	Promoters	T	Ensembl	NO	Own system	[70]
	Mouse/Rat							
	Dog/Chicken							
Pscan	Human	YES	Promoters	J/T	NO	NO	Own system	[71]
	Mouse							
	Drosophila							
	Arabidopsis							
	Yeast							
Contra	Human	YES	Full genome	J/T	phastCons/TBA	NO	Own system	[60]
	Mouse							
	Chicken							
	Xenopus							
	Zebrafish							
	*Drosophila*							
	*melanogaster*							
	Yeast							
TF-MAP alignments	Trained for vertebrates	YES	Promoters	J/T	Smith & Waterman (maps)	NO	GFF2PS	[47]
			Enhancers					
EEL	Trained for vertebrates	NO	Enhancers	J	Smith & Waterman	NO	NO	[72]
RELA	Trained for vertebrates	YES	Promoters	J/T	Smith & Waterman	NO	Own system	[73]
			Enhancers					
RSA-tools	Generic	YES	Promoters	-	Multiple approaches	NO	Own system	[29]
			Enhancers					
Footprinter	Trained for vertebrates	YES	Promoters	-	Footprinter	NO	Own system	[74]
			Enhancers					
Conreal	Trained for vertebrates	YES	Promoters	J/T	CONREAL	NO	Own system	[75]
			Enhancers		LAGAN			
					MAVID			
					BLASTZ			
TOUCAN	Trained for vertebrates and plants	YES	Full genome	J/T	Lagan/Avid/ BlastZ/Footprinter	NO	Own system	[76]

For each resource, we studied the following features: name, analyzed genomes, availability of the web server, class of genome sequences, catalogs of predictive models that are incorporated (J for Jaspar and T for Transfac), use of phylogenetical conservation information for evaluation of hits, use of chromatin profiles for reinforcing predictions, and the method selected to display the location of potential hits and reference.

It is likely that current predictors will include chromatin and epigenomics information in the near future to refine their predictive output; however, to our knowledge, the CBS platform of regulatory predictions is the first tool that integrates information on computationally identified TFBSs and histone modification marks to characterize *Drosophila* genomes. On the other hand, with the rapid sequencing of multiple genomes, a significant number of studies that characterize regulatory regions have introduced the evaluation of conservation across species as another discriminant factor to get rid of false positives. Although it is assumed that this information is valuable for this process in most scenarios, just a handful of currently available tools take advantage of this option to reduce the size of the set of predictions (CBS, Centipede [18], BLS [6], GenomeSurveyor [68], see Table 4).

It is assumed currently that not only promoter sequences but also distal enhancers and introns of genes may harbor functional binding sites. However, we still observed a bias in the number of applications exclusively implemented towards the characterization of promoter sequences (e.g. TFMExplorer [65], DoOPSearch [66], COTRASIF [67], Mapper [69], CoreTF [70], and Pscan [71], see Table 4). For this reason, tools such as CBS or GenomeSurveyor [68], which allow users to conduct virtual screenings on the full sequence of genomes, are much more informative. Finally, it is important to take into account the effort that non-expert users need to make to obtain the final results for a particular query. Although many approaches provide access to their maps of predictions through web servers that intuitively accept queries to output appropriate results (e.g. TFMExplorer [65], Mapper [69], and Pscan [71]), only CBS is able to automatically exchange information with popular genome browsers to reconstruct global prediction maps along the genome from custom tracks.

### Limitations of CBS predictions

Considering that informatics predictions offer information of limited value in certain situations, we would like to stress potential pitfalls that can affect the quality of CBS predictions: (i) poor specificity of published predictive models for some TFs due to the lack of biological information; (ii) uncompleteness of genome assemblies and the effect on the resulting inter-species alignments; (iii) strong sequence conservation sites that are not always functional because other elements, such as chromatin structure, can influence their activity; (iv) functional sites that are species-specific can be omitted when performing phylogenetic footprinting; (v) ChIP-seq experiments performed on heterogeneous cell populations might produce contradictory epigenomic profiles as a result of the overlap between distinct regulatory landscapes from different tissues; (vi) software for detection of regions enriched in ChIP-seq signals relies on the application of variable thresholds, which affects the final set of results on each case; and (vii) existence of alternative promoters and alternatively spliced forms that can harbor functional sites specific on certain isoforms. Taking into account these considerations and proceeding with caution in every situation, we believe that CBS predictions will constitute a highly valuable resource for researchers.

### Future development

Emerging high-throughput technologies are rapidly changing the class of regulatory information that is available to perform computational analysis of genomes. To appropriately evolve into this scenario, we are working on multiple lines of research to update CBS in the future: (a) incorporate published ChIP-seq profiles of TFs in *Drosophila* to evaluate the consistency of computational predictions; (b) include profiles for other histone marks that are thought to participate in gene transcription regulation (e.g. H3K9Ac); (c) integrate ChIP data and computational maps of Polycomb and Trithorax binding sites, which constitute the core of the regulatory machinery for many genes throughout the fly development; (d) display RNA-seq from modENCODE [77] on each developmental stage to inform about the expression of genes; (e) include experimental information on other *Drosophila* genomes when available; (f) integrate tools such as TF-map alignments [47] or EEL [72] to provide maps that harmonize predictions and experimental data; and (g) implement a mechanism to perform automatical updates of external repositories integrated in CBS.

## Conclusions

The CBS platform is an open resource developed to bridge the gap between experimental researchers and computational predictive methods. Access to this information is provided through a friendly and intuitive web interface, allowing users to easily gain knowledge. Importantly, flexibility of use in CBS does not require a limitation in the volume of information provided to users. In fact, we offer here the most comprehensive compilation of phylogenetically conserved binding sites and epigenomics predictions in the fruit fly genome published to date. In summary, we believe that CBS constitutes an excellent tool for assisting the experimental characterization of regulatory regions of *Drosophila*.

## Availability and requirements

Project name: CBS

Project home page: http://compfly.bio.ub.es/CBS/

Operating system(s): Platform independent

Programming language: PHP

License: Free

Any restrictions to use by non-academics: None

## Abbreviations

CBS: Conserved binding sites; TF: Transcription factor; TFBS: TF binding site; CRM: Cis-regulatory module; modENCODE: Model organism encyclopedia of DNA elements; ChIP-seq: Chromatin immunoprecipitation followed by massive DNA sequencing; H3K4Me1: Monomethylation of lys4 of histone H3; H3K27Ac: Acetylation of lys27 of histone H3; H3K27Me3: Trimethylation of lys27; H3K4Me3: Trimethylation of lys4 of histone H3.

## Competing interests

The authors declare that they have no competing interests.

## Authors’ contributions

EB and MC conceived the bioinformatics analysis. EB performed the bioinformatics experiments. EB and MC wrote the paper. All authors read and approved the final manuscript.

## Supplementary Material

Additional file 1 **List of modENCODE histone modification profiles along each developmental stage that are incorporated into the prediction of CBS enhancers.** For each ChIP-seq profile, the following information is given: histone mark, developmental stage, number of regions significantly enriched on this sample as compared to a control as reported by modENCODE, genome coverage, and the NCBI-GEO accession code. **Additional file 2.** List of combinations between modENCODE histone modification profiles along each developmental stage that are incorporated into the prediction of CBS enhancers. For each combination we show this information: combination of histone marks, developmental stage, genome coverage and percentage of H3K4Me1 regions that present intersection with the second mark. **Additional file 3.** List of combinations between modENCODE histone modification profiles along each developmental stage that are incorporated into the prediction of CBS enhancers after removing those regions overlapping RefSeq exons. For each combination, the set of histone marks, developmental stage, and genome coverage is given. **Additional file 4.** Visualization of CBS information on the modENCODE genome Browser. The following information is displayed (from top to bottom): FlyBase *en* gene annotation CBS predictions for several TFs that are known to participate in the regulation of this gene, REDfly experimental CRMs on this locus, BLS predictions, and ChIP-seq information about H3K4Me1, H3K27Ac, and H3K27Me3, as provided by modENCODE. **Additional file 5.** Dynamic regulatory pattern landscape along a genome region in embryos (12–16 h). The following information tracks are displayed along this fragment of 300 kb: (i) modENCODE H3K4Me1 ChIP-seq profile in red, H3K27Ac in blue, and H3K27Me3 in green; (ii) CBS evolutionarily conserved enhancers derived from previous profiles; active enhancers are highlighted in blue, and poised enhancers, in green; and (iii) RefSeq gene annotation and UCSC conservation tracks. **Additional file 6.** Characterizing putative enhancers in a gene-free region with CBS. A region of 50 kb in chromosome 2L that does not contain any RefSeq annotation is shown. The H3K4Me1/H3K4Me3 profiles, and the set of putative enhancers evolutionarily conserved predicted by CBS at this locus, are shown for embryos of 0–4 h and 12–16 h. **Additional file 7.** Conservation levels in TF binding regions of five different ChIP-seq experiments from the modENCODE project. Conservation was calculated as the average phastCons value along each hit, as reported by modENCODE. **Additional file 8.** Accuracy evaluation of CBS predictions on modENCODE GAF/Trl ChIP-seq binding regions. At the top, the distribution of successfully identified ChIP-seq sites for CBS predictions is shown, taking into account different levels of sequence conservation. At the bottom, the ratio between the number of predictions and the number of successfully identified ChIP-seq sites for the same conservation thresholds is given. **Additional file 9.** Evaluation of CBS annotations in the *even-skipped* gene stripe 2 enhancer (GenBank: AF042709, dm3: chr2R:5865217–5865890). The following binding sites have been experimentally validated [78]: bicoid (+138, +159, +310, +403, +521), hunchback (+496, +578, +661), and Kruppel (+3, +139, +327, +521, +571, +615). At the top, we show the UCSC multiz15way conservation track. For each TF, we display the MatScan matrix score, CBS annotations, and experimental sites. This figure was graphically customized from original CBS results, incorporating the location of experimentally validated sites and the score of the weight matrix predictions into the final picture. Click here for file
