# Supplementary material for: CBS: an open platform that integrates predictive methods and epigenetics information to characterize conserved regulatory features in multiple Drosophila genomes
Source: BMC Genomics. 2012 Dec 10;13:688. doi: 10.1186/1471-2164-13-688 (PMC3564944; doi:10.1186/1471-2164-13-688)
Supplement: Additional file 1 — List of modENCODE histone modification profiles along each developmental stage that are incorporated into the prediction of CBS enhancers. For each ChIP-seq profile, the following information is given: histone mark, developmental stage, number of regions significantly enriched on this sample as compared to a control as reported by modENCODE, genome coverage, and the NCBI-GEO accession code. Additional file 2. List of combinations between modENCODE histone modification profiles along each developmental stage that are incorporated into the prediction of CBS enhancers. For each combination we show this information: combination of histone marks, developmental stage, genome coverage and percentage of H3K4Me1 regions that present intersection with the second mark. Additional file 3. List of combinations between modENCODE histone modification profiles along each developmental stage that are incorporated into the prediction of CBS enhancers after removing those regions overlapping RefSeq exons. For each combination, the set of histone marks, developmental stage, and genome coverage is given. Additional file 4. Visualization of CBS information on the modENCODE genome Browser. The following information is displayed (from top to bottom): FlyBase en gene annotation CBS predictions for several TFs that are known to participate in the regulation of this gene, REDfly experimental CRMs on this locus, BLS predictions, and ChIP-seq information about H3K4Me1, H3K27Ac, and H3K27Me3, as provided by modENCODE. Additional file 5. Dynamic regulatory pattern landscape along a genome region in embryos (12–16 h). The following information tracks are displayed along this fragment of 300 kb: (i) modENCODE H3K4Me1 ChIP-seq profile in red, H3K27Ac in blue, and H3K27Me3 in green; (ii) CBS evolutionarily conserved enhancers derived from previous profiles; active enhancers are highlighted in blue, and poised enhancers, in green; and (iii) RefSeq gene annotation and UCSC conservation tracks. Addition [file 1471-2164-13-688-S1.pdf]

MARK	STAGE	# REGIONS	COVERAGE	NCBI-GEO
H3K4Me1	E0-4h	6158	7349480	GSM401409
	E-4-8h	62	755934	GSM401406
	E-8-12h	5260	9973919	GSM432593
	E12-16h	17047	24152551	GSM432591
	E16-20h	12323	22684047	GSM401403
	E20-24h	9998	5139712	GSM439464
	L1	1409	2670631	GSM432588
	L2	10818	14740000	GSM401421
	L3	379	581641	GSM401418
	Pupae	6854	10054759	GSM401415
	Adult (M)	1117	1902408	GSM401411
	Adult (F)	7489	3035652	GSM439465
H3K27Ac	E0-4h	3654	9129325	GSM401407
	E-4-8h	1119	5150316	GSM401404
	E-8-12h	4402	9246096	GSM432583
	E12-16h	10592	14482029	GSM432582
	E16-20h	11250	17537981	GSM401401
	E20-24h	7500	14746489	GSM401423
	L1	1984	3402719	GSM432581
	L2	3757	9941986	GSM401419
	L3	4160	10685090	GSM401416
	Pupae	3113	6837528	GSM401413
	Adult (M)	1	7784	GSM401410
	Adult (F)	0	0	GSM401412
H3K27Me3	E0-4h	363	17294692	GSM439448
	E-4-8h	242	145827	GSM439447
	E-8-12h	230	14084439	GSM439446
	E12-16h	147	10488228	GSM439445
	E16-20h	292	19437192	GSM439444
	E20-24h	144	10727412	GSM439443
	L1	208	17266388	GSM439442
	L2	137	11504372	GSM439441
	L3	174	13138335	GSM439440
	Pupae	276	24642554	GSM439439
	Adult (M)	123	13106788	GSM439438
	Adult (F)	207	9802101	modMine
H3K4Me3	E0-4h	6806	6293472	GSM400656
	E-4-8h	4234	8051158	GSM400674
	E-8-12h	4456	9488107	GSM432585
	E12-16h	5104	9329454	GSM432580
	E16-20h	9466	14872146	GSM400658
	E20-24h	10915	15795516	GSM400672
	L1	5517	10349253	GSM400662
	L2	4482	12295219	GSM400668
	L3	5100	12469370	GSM400660
	Pupae	5641	8570627	GSM400664
	Adult (M)	5886	6065409	GSM400666
	Adult (F)	4775	10989238	GSM400670

Additional Data File 1

MARK	STAGE	COVERAGE	% H3K4Me1
H3K4Me1	E0-4h	7349480	
	E-4-8h	755934	
	E-8-12h	9973919	
	E12-16h	24152551	
	E16-20h	22684047	
	E20-24h	5139712	
	L1	2670631	
	L2	14740000	
	L3	581641	
	Pupae	10054759	
	Adult (M)	1902408	
	Adult (F)	3035652	
+H3K27Ac	E0-4h	3233173	40%
	E-4-8h	584624	80%
	E-8-12h	5291313	50%
	E12-16h	10348157	40%
	E16-20h	12664176	60%
	E20-24h	3351206	70%
	L1	914947	30%
	L2	908429	10%
	L3	354022	60%
	Pupae	2662220	30%
	Adult (M)	0	0
	Adult (F)	0	0
+H3K27Me3	E0-4h	1312172	20%
	E-4-8h	145827	20%
	E-8-12h	2674422	30%
	E12-16h	3783299	20%
	E16-20h	4099211	20%
	E20-24h	428890	10%
	L1	264436	10%
	L2	721609	0%
	L3	34379	10%
	Pupae	1054728	10%
	Adult (M)	38522	0%
	Adult (F)	54155	0%
-H3K4Me3	E0-4h	4742292	60%
	E-4-8h	640155	80%
	E-8-12h	6234863	60%
	E12-16h	18616083	80%
	E16-20h	10724227	50%
	E20-24h	2371026	50%
	L1	1077711	40%
	L2	7582856	50%
	L3	175723	30%
	Pupae	5977372	60%
	Adult (M)	1399119	70%
	Adult (F)	1452727	50%

Additional Data File 2

MARK	STAGE	COVERAGE
H3K4Me1	E0-4h	3845858
	E-4-8h	683963
	E-8-12h	6064648
	E12-16h	17527714
	E16-20h	12074836
	E20-24h	2911513
	L1	617186
	L2	4043032
	L3	346357
	Pupae	2370672
	Adult (M)	167230
	Adult (F)	1439674
+H3K27Ac	E0-4h	1359424
	E-4-8h	496020
	E-8-12h	2882917
	E12-16h	6432852
	E16-20h	5904232
	E20-24h	1746187
	L1	167689
	L2	375341
	L3	210261
	Pupae	716887
	Adult (M)	0
	Adult (F)	0
+H3K27Me3	E0-4h	1115641
	E-4-8h	128034
	E-8-12h	2258056
	E12-16h	3463175
	E16-20h	3099661
	E20-24h	321911
	L1	66958
	L2	308834
	L3	23281
	Pupae	350039
	Adult (M)	4413
	Adult (F)	45577
-H3K4Me3	E0-4h	3049279
	E-4-8h	580287
	E-8-12h	4867724
	E12-16h	15368363
	E16-20h	8133091
	E20-24h	2081899
	L1	399968
	L2	2516164
	L3	143639
	Pupae	1621861
	Adult (M)	112599
	Adult (F)	1009369

Additional Data File 3

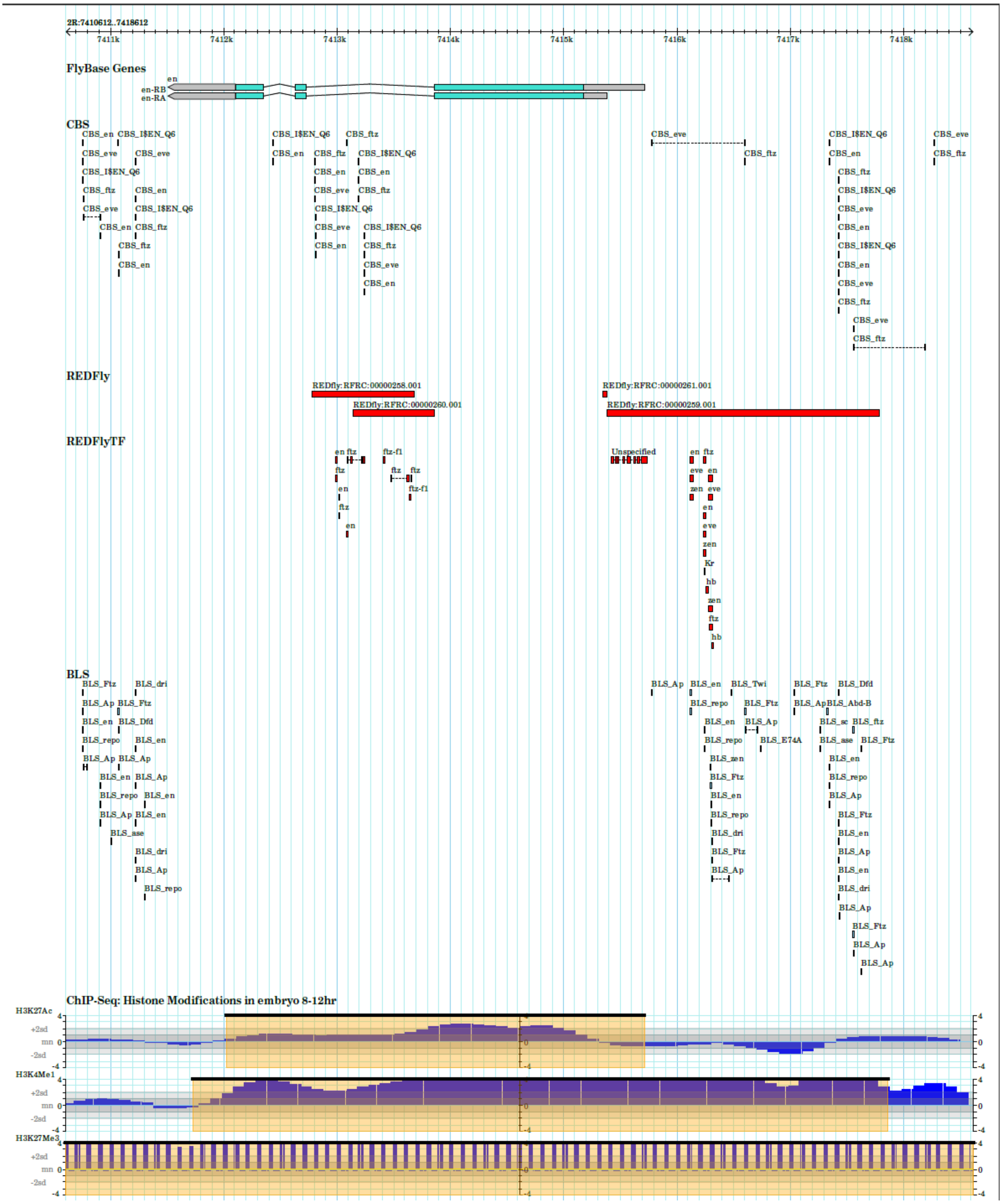

Additional Data File 4

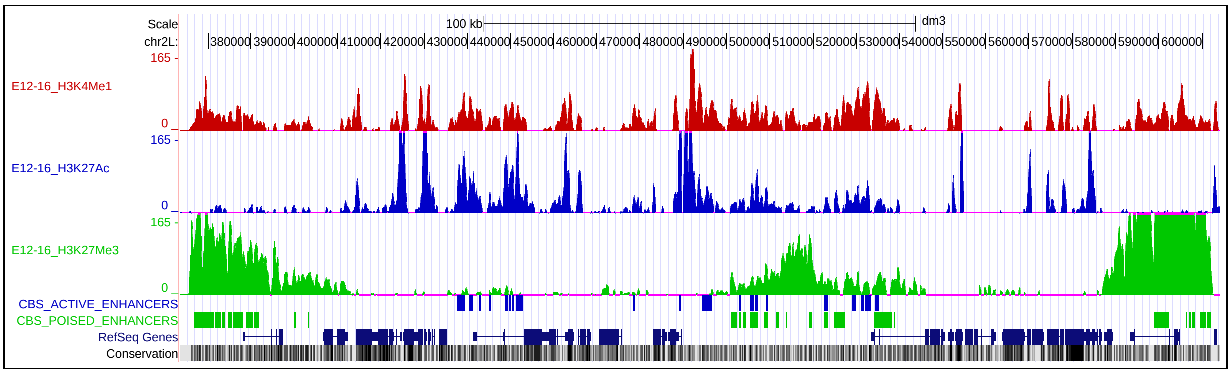

Additional Data File 5

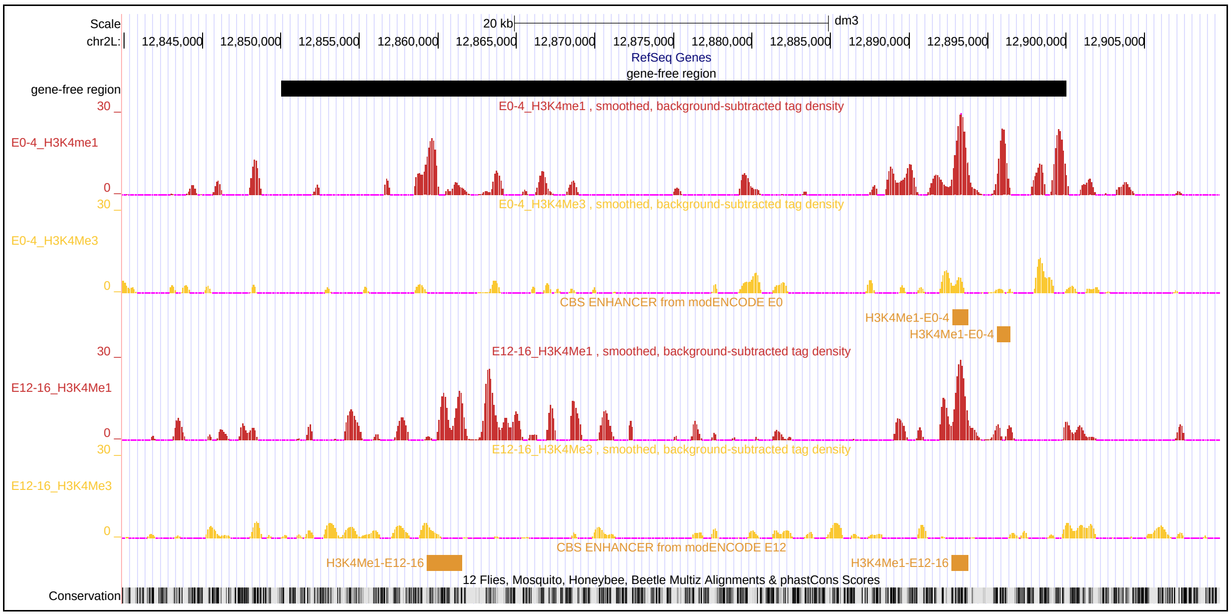

Additional Data File 6

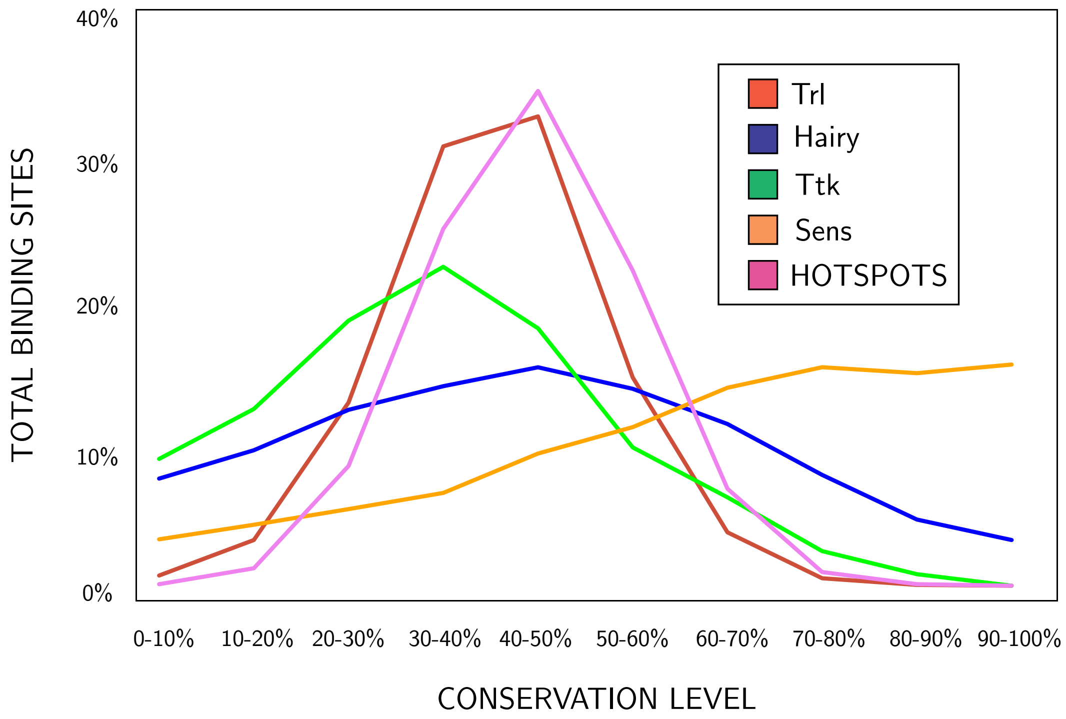

Additional Data File 7

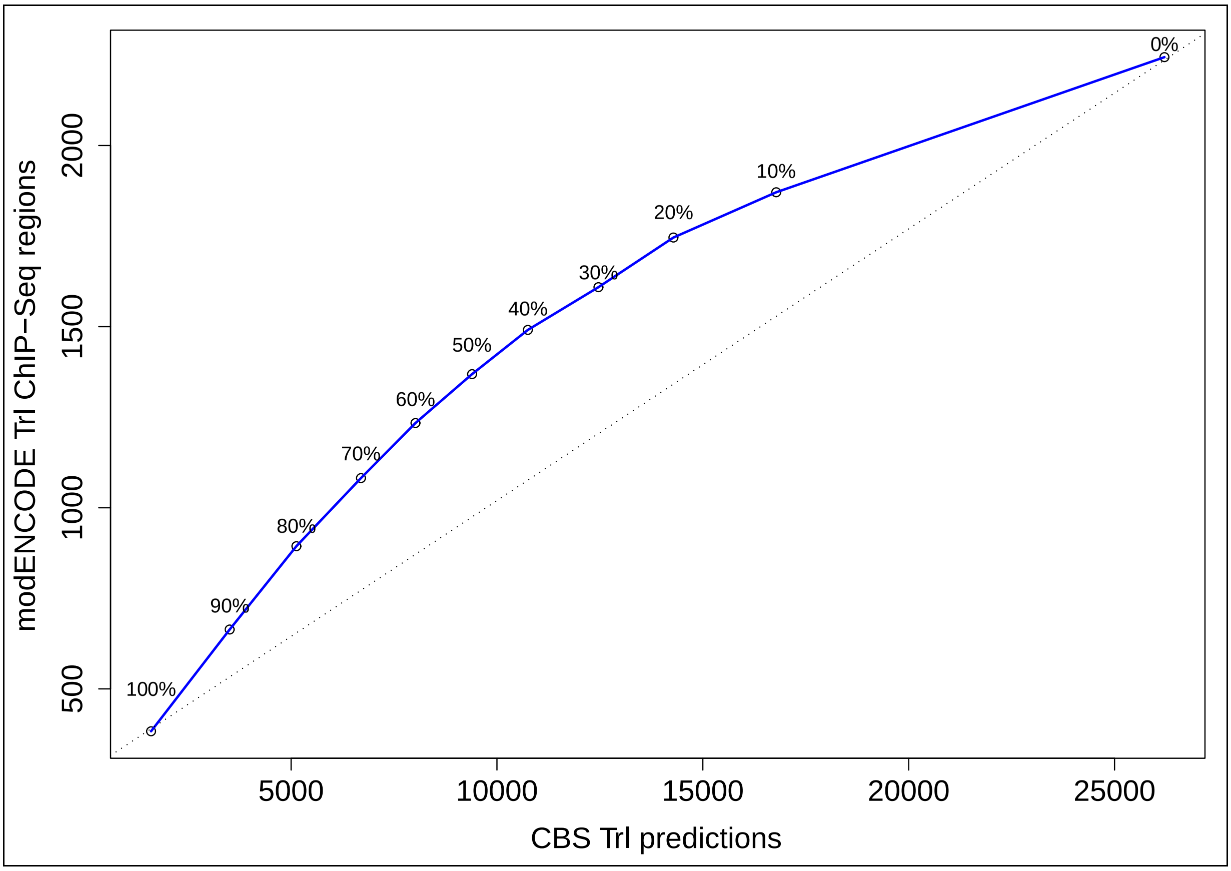

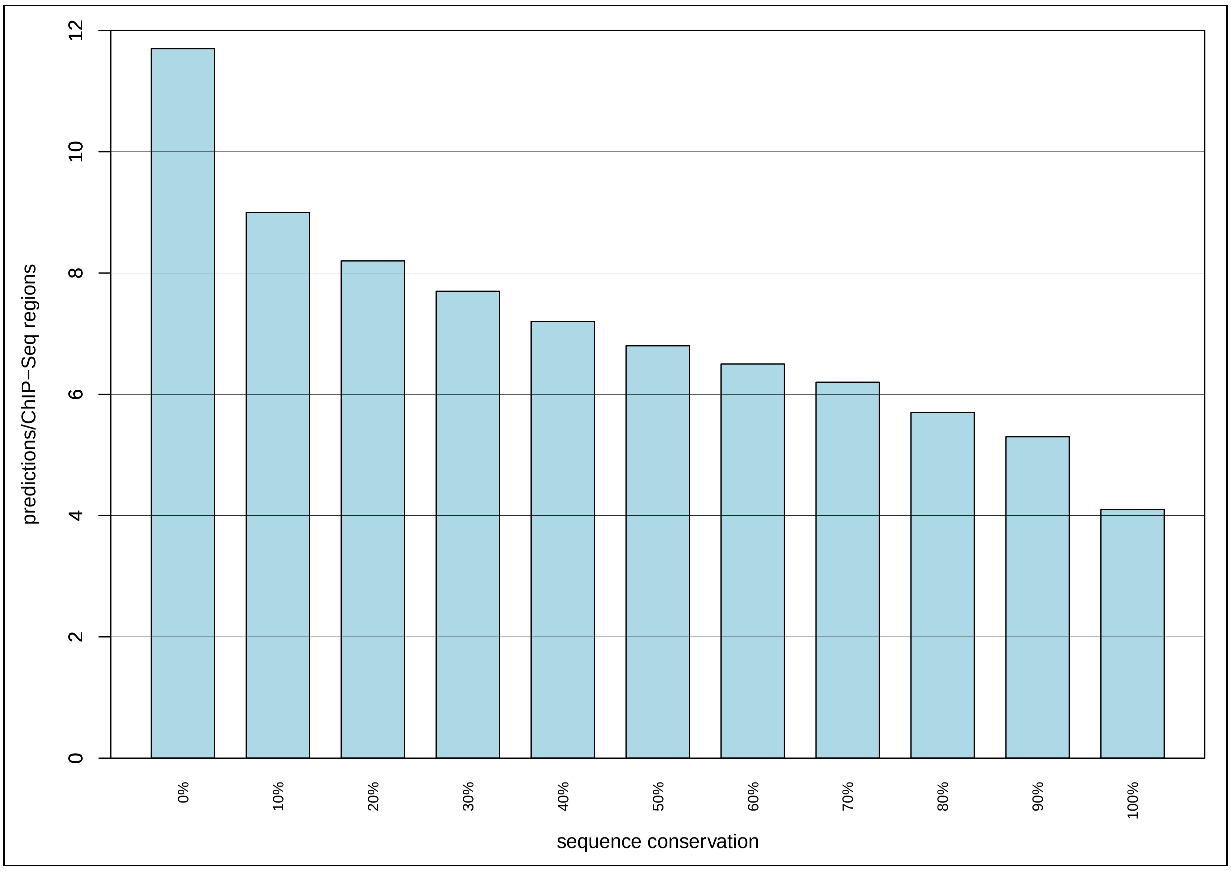

Additional Data File 8

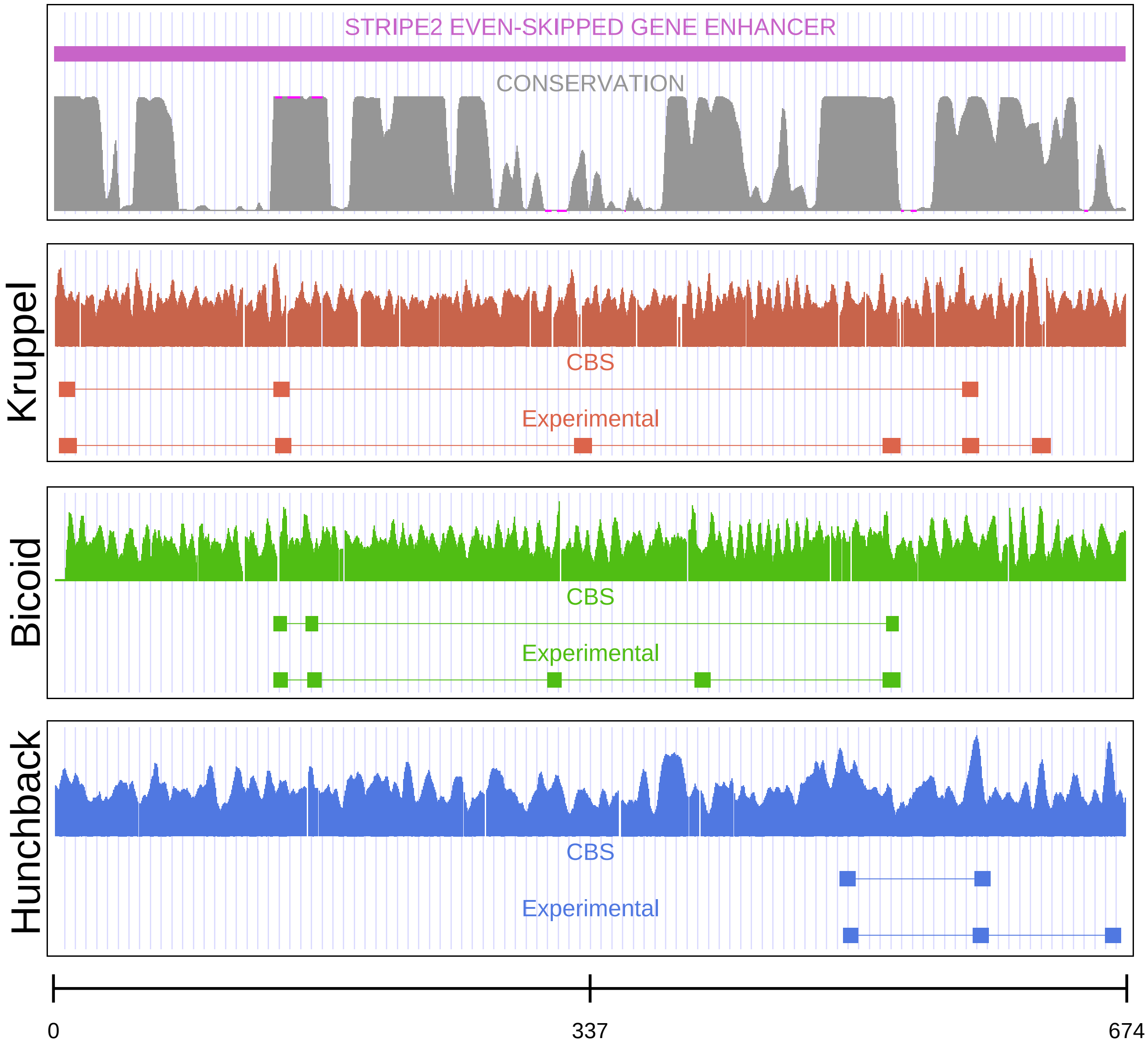

Additional Data File 9
